# hsa_circ_0000092 promotes hepatocellular carcinoma progression through up‐regulating HN1 expression by binding to microRNA‐338‐3p

**DOI:** 10.1111/jcmm.15010

**Published:** 2020-02-20

**Authors:** Jian Pu, Jianchu Wang, Wenchuan Li, Yuan Lu, Xianjian Wu, Xidai Long, Chunying Luo, Huamei Wei

**Affiliations:** ^1^ Department of Hepatobiliary Surgery Affiliated Hospital of Youjiang Medical University for Nationalities Baise China; ^2^ Graduate College of Youjiang Medical University for Nationalities Baise China; ^3^ Department of Pathology Affiliated Hospital of Youjiang Medical University for Nationalities Baise China

**Keywords:** competing endogenous RNA, haematopoietic‐ and neurologic‐expressed sequence 1, hepatocellular carcinoma, hsa_circ_0000092, microRNA‐338‐3p

## Abstract

Circular RNAs (circRNAs) have been identified in diverse cancers for their role in regulating multiple cellular processes by antagonizing microRNAs (miRNAs or miRs). However, the role of circRNA hsa_circ_0000092 in hepatocellular carcinoma (HCC) still remains enigmatic. Therefore, we aimed to investigate the specific mechanism of hsa_circ_0000092 in HCC. Differentially expressed circRNAs associated to HCC were initially analysed. The expression of hsa_circ_0000092, miR‐338‐3p and HN1 in HCC tissues and cell lines was examined. Next, the interaction among hsa_circ_0000092, miR‐338‐3p and HN1 was determined by dual‐luciferase reporter, RNA pull‐down and northern blot assays. Subsequently, a series of mimic, inhibitor or siRNA plasmids were delivered into HCC cells to validate the effects of hsa_circ_0000092, miR‐338‐3p and HN1 in controlling cell proliferation, migration, invasion and angiogenesis in vitro. Furthermore, the role of hsa_circ_0000092 in tumour growth of HCC in vivo was assessed with hsa_circ_0000092 depleted with siRNA. The hsa_circ_0000092/miR‐338‐3p/HN1 axis was predicted to participate in the development of HCC. hsa_circ_0000092 and HN1 were highly expressed while miR‐338‐3p was poorly expressed in HCC tissues and cell lines. hsa_circ_0000092 could competitively bind to miR‐338‐3p to up‐regulate HN1 expression. Moreover, depleted hsa_circ_0000092 or elevated miR‐338‐3p was shown to suppress HCC cell proliferation, migration, invasion and angiogenesis in vitro via down‐regulation of HN1. Furthermore, silencing hsa_circ_0000092 was demonstrated to suppress tumour growth in HCC in vivo. The results of this study suggested that hsa_circ_0000092 impaired miR‐338‐3p‐mediated HN1 inhibition to aggravate the development of HCC, indicating that hsa_circ_0000092 is a potential candidate marker and therapeutic target for HCC.

## 1 INTRODUCTION

Hepatocellular carcinoma (HCC), also known as primary liver cancer, ranks fifth among the most prevalent cancers in males and seventh in females around the world^1^ and is associated with a high mortality rate, especially in China.^2^ HCC is primarily caused by hepatitis B and hepatitis C infections as well as other numerous risk factors, such as drinking, smoking, exposure to aflatoxin and obesity.^3^ HCC therapies include surgical options, radiological therapies, molecule‐targeted and systemic chemical therapies^4^, ^5^; however, the treatment of HCC still remains a big challenge due to liver dysfunction and poor chemosensitivity.^6^ In addition, despite improvements in surveillance, patients with HCC still suffer from a poor survival rate since patients with HCC are always diagnosed at an advanced stage, which is caused by rapid proliferation and metastasis of HCC tumour cells.^7^ Thus, finding a possible molecular target of cell biological functions in HCC might be of highest clinical significance in finding a novel treatment for the disease.

Recent evidence has identified aberrant expression of circular RNAs (circRNAs) in HCC and that circRNAs are closely associated with the development of HCC.^8^ Various circRNAs are reported to regulate several biological processes associated with the progression of HCC and may serve as prognostic indicators or therapeutic targets of HCC.^9^ hsa‐circ‐0001649, for instance, has been highlighted as an intriguing target for HCC due to its involvement in the metastasis and tumorigenesis of HCC.^10^ Also, in our study, hsa_circ_0000092 was predicted to be differentially expressed in HCC and plays a role in the progression of HCC. Moreover, circRNAs regulate cancers, including HCC, by acting as competing endogenous RNAs (ceRNAs), which serve as microRNA (miRNA or miR) sponges.^11^ miR‐338‐3p, the antisense of miR‐338 gene on chromosome 17, appears to be poorly expressed in liver cancer cells, and its overexpression inhibits cell migration and proliferation in liver cancer.^12^ Down‐regulation of miR‐338‐3p is also found in HCC where it also acts as a tumour suppressor of HCC.^13^ An online prediction website RNA22 (https://cm.jefferson.edu/rna22/) predicted that miR‐338‐3p could target haematopoietic‐ and neurologic‐expressed sequence 1 (HN1) in HCC. HN1, initially found in mouse embryonic tissues,^14^ participates in many cellular processes in various diseases.^15^ For instance, HN1 correlates with a poor prognosis of patients with HCC and its knockdown is capable of curtailing cell growth and migration potentials partly by down‐regulation of c‐Met.^16^ Based on the above findings, we suggested that the hsa_circ_0000092/miR‐338‐3p/HN1 axis might be involved in the development of HCC. To verify this, we performed a series of in vitro and in vivo experiments to elucidate the underlying mechanisms by which the hsa_circ_0000092/miR‐338‐3p/HN1 axis regulates the progression of HCC.

## 2 MATERIALS AND METHODS

### 2.1 Ethics statement

This study protocol was approved by the Ethics Committee and the Experimental Animal Ethics Committee affiliated with the Affiliated Hospital of Youjiang Medical University for Nationalities. Written informed consent was obtained from all participants or their relatives prior to enrolment. All experimental methods abided by the *Declaration of Helsinki*. The animal experiments strictly adhered to the principle of minimizing the pain, suffering and discomfort of experimental animals.

### 2.2 Patient enrolment

A total of 40 patients with HCC (28 males and 12 females) treated at the Affiliated Hospital of Youjiang Medical University for Nationalities from June 2015 to January 2018 were selected for this study. Among these patients, 11 were classified as stage I, 15 as stage II and 14 as stage III. Patients participating in the study had not received any anti‐tumour treatment prior to the surgery.

### 2.3 Cell culture

Human HCC cell lines (Hep3B, LM3, MHCC97L, SK‐hep1 and HepG2), normal human liver cell line (THLE‐2) and human umbilical vein endothelial cells (HUVECs), purchased from American Type Culture Collection (ATCC), were inoculated into the Roswell Park Memorial Institute (RPMI) 1640 medium (11875093, Gibco‐BRL, Invitrogen Corporation) supplemented with 10% foetal bovine serum (FBS; 10100147, Gibco‐BRL) and penicillin/streptomycin (100 units per mL of medium; 15140122, Gibco‐BRL, Invitrogen Corporation) at 37°C with 5% CO_2_.

### 2.4 Cell stimulation

Based on the instructions of the manufactures, 1 × 10^6^ cells were treated with plasmids of 50 nmol/L of miR‐338‐3p mimic, miR‐338‐3p inhibitor or the corresponding negative control (NC) (Shanghai Sangon Biotechnology Co., Ltd.) in 1 μL Lipofectamine™ 2000 reagent (11668019, Invitrogen). Small interfering RNAs (siRNAs) from the Sigma Mission RNAi shRNA vector library (Sigma‐Aldrich Chemical Company) were used to suppress the expression of hsa_circ_0000092 and HN1. siRNA delivery was performed in accordance with the instructions of Lipofectamine™ RNA iMAX reagent (13778030, Invitrogen).

### 2.5 Fluorescence in situ hybridization (FISH)

The subcellular localization of hsa_circ_0000092 was detected using FISH according to the instructions of Ribo™ lncRNA FISH Probe Mix (Red) (Guangzhou RiboBio Co., Ltd.). In brief, the cultured HCC cells in a 6‐well plate were fixed with 4% paraformaldehyde solution (1 mL) upon reaching 80% confluence, followed by treatment with protease K (2 μg/mL). Afterwards, the cells were pre‐hybridized with 250 μL solution for 1 hour and then hybridized with 250 μL solution containing hsa_circ_0000092 probe (300 ng/mL). The nucleus was stained with 4′,6′‐diamidino‐2‐phenylindole diluted by phosphate‐buffered saline containing 0.1% Tween‐20 (PBST) for 5 minutes and photographed in 5 randomly selected visual fields under a fluorescence microscope (Olympus Optical Co., Ltd).

### 2.6 Dual‐luciferase reporter assay

The full‐length of hsa_circ_0000092 and the 3′untrasnlated region (3′UTR) of HN1 mRNA as well as the mutated sequence of their independent binding sites of miR‐338‐3p (hsa_circ_0000092‐mutant [mut], hsa_circ_0000092‐wild‐type [wt], HN1‐mut and HN1‐wt) were cloned into the psiCheck2 plasmid as the luciferase reporter gene. NC mimic and miR‐338‐3p mimic were cotransfected with luciferase report vectors into LM3 and SK‐hep1 cells, respectively. Luciferase activity was measured in the presence of miR‐338‐3p mimic using the dual‐luciferase detection kits (Promega). The firefly luciferase activity was measured using the Dual‐luciferase Reporter Assay System (Promega) with renilla luciferase activity as the internal control.

### 2.7 Reverse transcription quantitative polymerase chain reaction (RT‐qPCR)

Total RNA was regularly extracted from tissues or cell lines according to the instructions of the TRIzol reagent (15596‐018). The primers of hsa_circ_0000092, miR‐338‐3p and HN1 (Table 1) were designed using Primer 5.0 software and synthesized by Sangon Biotech Co., Ltd. RT‐qPCR was performed using the TaqMan probe method according to the protocols of the kits (MBI). The reaction was detected using the quantitative PCR reaction system (Bio‐Rad iQ5; Bio‐Rad). U6 was used as the internal control for miR‐338‐3p and glyceraldehyde‐3‐phosphate dehydrogenase (GAPDH) for hsa_circ_0000092 and HN1. The relative expression of the target gene was calculated based on the 2^−ΔΔCt^ method.

**TABLE 1:** Primer sequences for reverse transcription quantitative polymerase chain reaction

Gene	Sequences
hsa_circ_0000092	F: 5′‐TAGCAGTTCCCCAATCCTTG‐3′
	R: 5′‐CACAAATTCCCATCATTCCC‐3′
miR‐338‐3p	F: 5′‐TTAGTG TACCAGCCAT‐3′
	R: 5′‐GAATGCGGGAGCGAA‐3′
miR‐188‐3p	F: 5′‐ATTATTGGCTCCCACATGCAGGG‐3′
	R: 5′‐ATCCAGTGCAGGGTCCGAGG‐3′
miR‐513a‐5p	F: 5′‐AGGAGCTCGAGACCATCCTG‐3′
	R: 5′‐TAGAGTTTAAACATTTTTA‐3′
miR‐587	F: 5′‐CCAGGCAAGAGAGAGTTGCTG‐3′
	R: 5′‐AGTCACAGGTGCAGACACATT‐3′
HN1	F: 5′‐ATAGCTCCCGAGTTTTGCG‐3′
	R: 5′‐TTGGCCCAAGAAGCTTGA‐3′
U6	F: 5′‐AGCCCGCACTCAGAACATC‐3′
	R: 5′‐GCCACCAAGACAATCATCC‐3′
GAPDH	F: 5′‐CGCTCTCTGCTCCTCCTGTTC‐3′
	R: 5′‐ATCCGTTGACTCCGACCTTCAC‐3′

Abbreviations: F, forward; GAPDH, glyceraldehyde‐3‐phosphate dehydrogenase; HN1, haematopoietic‐ and neurologic‐expressed sequence 1; miR, microRNA; R, reverse.

### 2.8 Western blot analysis

Western blot analysis was performed as described previously^17^ with the following antibodies: HN1 (ab247137, rabbit antibody, 1:2500, Abcam Inc), GAPDH (ab9485, rabbit antibody, 1:2000, Abcam Inc) and secondary goat anti‐rabbit immunoglobulin G (ab6721, 1:20 000, Abcam Inc). The grey value of protein expression was measured using the NIH software Image J.

### 2.9 RNA pull‐down assay

The biological coupling probe specifically binding to hsa_circ_0000092 was designed. Approximately 1 × 10^7^ cells were washed with pre‐cooled PBS, lysed and allowed to stand at room temperature with the addition of biological coupling probe for 2 hours. After that, cells were treated with streptavidin‐coated magnetic beads (Life Technologies) for 4 hours to isolate the biotin‐conjugated RNA probe. The beads were washed with lysis buffer, and RNA was extracted using the TRIzol method, and target RNAs were analysed using RT‐qPCR.^18^

A total of 2 × 10^6^ cells were treated with 50 μmol/L biotin‐coupled miRNA in 50% lysis buffer. After 24 hours, cells were washed with PBS and lysed. Washed streptavidin‐coated magnetic beads (50 μL) were blocked for 2 hours and added into the reaction tubes to pull down the miRNA. The tubes were then incubated at a low speed for 4 hours, and the beads were washed with lysis buffer for 5 times, after which the miRNA was extracted using TRIzol LS (Thermo Fisher Scientific). The extracted RNA was analysed by RT‐qPCR and agarose gel.^18^

### 2.10 Northern blot analysis

RNA was extracted using the RNA extraction kit (R0026, Beyotime Biotechnology Co.) under RNase‐free conditions and subjected to formaldehyde denaturing gel electrophoresis, followed by ethidium bromide staining. The gel was illuminated using the ultraviolet projector (ZF‐90, Shanghai Jihui scientific analysis instrument Co. Ltd.). The remaining gel was subsequently immersed in 0.05 mol/L NaOH/1.5 mol/L NaCl to partially hydrolyse the RNA, followed by immersion in 0.5 mol/L Tris·C1 (pH = 7.4)/1.5 mol/L NaCl and then 20× silica sodium carbonate. RNA‐specific probe was used for mRNA hybridization, and the membrane was washed to remove the non‐specific binding hybridization. After developing or visualization, the hybridization signal was analysed and the intensity of the signals was compared to assess the expression of RNA.

### 2.11 5‐Ethynyl‐2'‐deoxyuridine (EdU) assay

EdU assay was conducted in strict accordance with the protocols of the Cell‐Light EdU DNA Cell Proliferation Kit (R11056.5, RiboBio company). Cells (2 × 10^4^) were inoculated into a 6‐well plate and cultured for 48 hours, after which the cells continued to be cultured for 2 hours with the medium replaced by 50 μmol/L EdU‐mixed medium. After fixing by 4% paraformaldehyde solution, cells were incubated with 0.5% Triton X‐100 penetrant, followed by the addition of 500 μL 1× Apollo®. After being washed with 0.5% Triton X‐100 penetrant, the cells were resuspended in PBS and observed under a fluorescence microscope.^19^

### 2.12 Colony formation assay

The monolayer cells at logarithmic growth phase were trypsinized and suspended in RPMI 1640 medium containing 10% foetal bovine serum. A total of 200 cells were inoculated into the culture dish with 10 mL pre‐heated culture liquid and cultured at 37°C with 5% CO_2_ and saturated humidity for 2‐3 weeks. With the removal of supernatant, the cells were rinsed twice with PBS and fixed with 5 mL methanol for 15 minutes. Next, the cells were stained with Giemsa staining liquid for 10‐30 minutes, washed with tap water to remove the staining liquid and then air‐dried. The number of colonies with more than 10 cells was counted under a low magnification microscope to calculate the colony formation rate.

### 2.13 Scratch test

Cells were inoculated into a 6‐well plate at a density of 2.5 × 10^4^ cells/cm^2^. After 24 hours of culture, the culture medium was removed and scratches were made. The wound healing of cells at the same location was observed at 0 hour and 48 hours. Three duplicated wells were set, and the migration ability of the HCC cells was expressed as the relative scratch width: relative scratch width = (the number of cells in the scratch zone at 48 hours − the number of cells in the scratch zone at 0 hour)/the number of cells in the scratch zone at 0 hour × 100%.

### 2.14 Transwell assay

The Transwell chambers were put into 24‐well plates, and the apical chamber of the basement membrane was coated with Matrigel diluent (1:8, 40111ES08, Shanghai Yeasen Biotechnology Co., Ltd.). A total of 200 μL cell suspension was added to Matrigel‐coated apical chamber and 600 μL RPMI 1640 medium containing 20% FBS was added to the basolateral chamber. The cells in the internal apical chamber were stained with 0.5% methanol‐prepared crystal violet solution for 15 minutes. After that, the cells were observed under an inverted microscope (XDS‐800D, Shanghai Caikon Optical Instrument Co., Ltd.) in 5 randomly selected visual fields and images were captured (200×). The transmembrane cells were counted, and three duplicate wells were set.

### 2.15 Vessel‐like tube formation in vitro

Culture medium of HCC cells in each group was added to the culture medium of HUVECs, and then, cells were incubated at 37°C with 5% CO_2_. After 9‐10 hours of incubation, the tube formation rate was determined using an inverted microscope (100×) to count the number of tubes in randomly selected visual fields.

### 2.16 Matrigel plug assay in vivo for detection of angiogenesis

HCC cells manipulated with si‐hsa_circ_0000092, miR‐338‐3p mimic, or combined si‐hsa_circ_0000092 and miR‐338‐3p inhibitor were co‐cultured with HUVECs and then were inoculated into the mice. Mice inoculated with HUVEC suspension were used as control. After 3 days of inoculation, the mice were injected with 500 μL Matrigel supplemented with vascular endothelial growth factor (VEGF, 250 ng/mL) in the abdomen, followed by 10 days of culture. After that, the mice were killed and the Matrigel was taken out. Next, the Matrigel was fixed overnight in 100 mL/L formalin solution containing PBS prepared using 25 mL/L glutaraldehyde, paraffin‐embedded, and sliced into sections for Masson staining. Afterwards, the Matrigel was observed under an inverted microscope in 5 randomly selected visual fields and the number of vessels was counted.

### 2.17 In situ hybridization (ISH)

The expression of miR‐338‐3p in HCC and adjacent normal tissues was analysed using the digoxin‐labelled miR‐338‐3p probe based on ISH (Shanghai Outdo Biotech Co., Ltd.). The hybridization was visualized and observed under an optical microscope or an electron microscope.

### 2.18 Immunohistochemistry (IHC)

Tissue samples were sliced into 5‐μm sections and incubated overnight at 4°C with the addition of the following primary antibodies: rabbit polyclonal antibody against HN1 (ab126705, 1:200), mouse antibody against proliferating cell nuclear antigen (PCNA, ab29, 1:10 000), rabbit polyclonal antibody against matrix metalloproteinase 2 (MMP2) (ab37150, 2‐4 µg/mL), rabbit polyclonal antibody against MMP9 (ab38898, 1:1000) and rabbit monoclonal antibody against VEGF (ab32152, 1:250). These antibodies were all purchased from Abcam Inc. Further, the sections were incubated with biotin‐labelled secondary goat anti‐rabbit immunoglobulin G (IgG) at 37°C for 20 minutes, developed using diaminobenzidine and counterstained using haematoxylin. The number of positive cells in 5 randomly selected visual fields was counted under a microscope.

### 2.19 Xenograft tumours in nude mice

A total of 30 male athymic BALB/c nude mice (aged 4‐6 weeks old and weighing 18‐24 g) purchased from Hunan SJA Laboratory Animal Co., Ltd. were reared under specific pathogen‐free conditions. LM3 cells stably transfected with NC or si‐hsa_circ_0000092 were made into cell suspension and then inoculated subcutaneously into the nude mice at a density of 1 × 10^6^ cells/mL. Tumour volume was monitored once a week. The length (*a*) and width (*b*) of the transplanted tumour were measured with a vernier calliper every week. The tumour volume (*V*) was calculated using the formula: *V* = (*a* × *b* ^2^)/2. The nude mice were then killed at the 5th week, and the tumour tissues were resected.

### 2.20 Statistical analysis

All data were processed using the SPSS 21.0 statistical software (IBM Corp. Armonk, NY, USA). The measurement data were expressed as mean ± standard deviation. The data conforming to normal distribution and homogeneity of variance between two groups were analysed using unpaired *t* test, and the data among multiple groups were analysed using one‐way analysis of variance (ANOVA), followed by Tukey's post hoc test. Data at different time‐points were analysed using repeated measures ANOVA, followed by Tukey's post hoc test. A value of *P* < .05 was considered to be indicative of statistical significance.

## 3 RESULTS

### 3.1 In silico analysis predicting the differentially expressed genes and their molecular interactions in HCC

**FIGURE 1:**
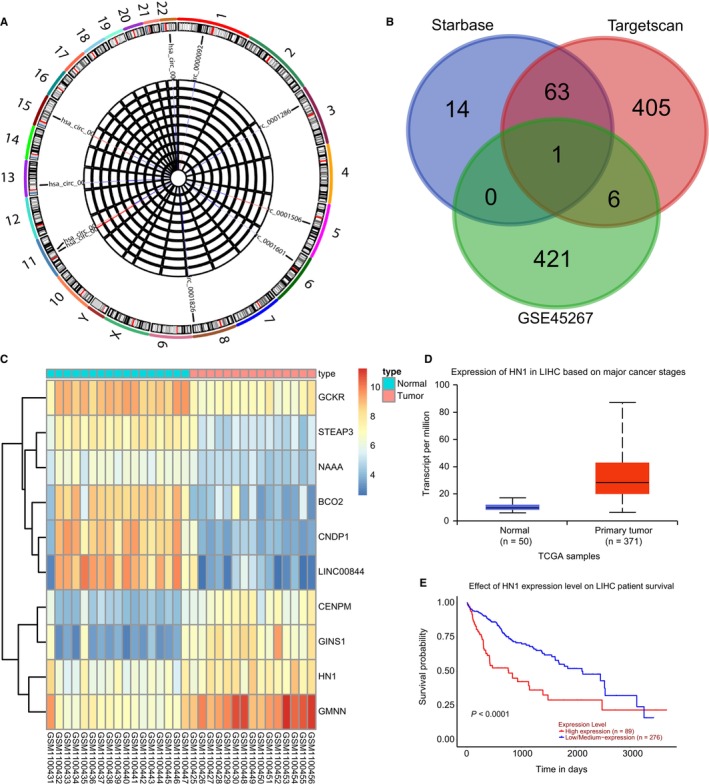
circRNA, miRNA and mRNA expression profiles in HCC. A, A heat map of the top 10 differentially expressed circRNAs in GSE94508 and expression of hsa‐miR‐188‐3p, hsa‐miR‐338‐3p, hsa‐miR‐513a‐5p and hsa‐miR‐587 in HCC and adjacent normal tissues. B, Intersection of up‐regulated genes in GSE45267 and the predicted results from the starbase and targetscan databases. C, A heat map of the top 10 differentially expressed genes in GSE45267. D, Expression of HN1 in HCC tissues retrieved from the TCGA database. E, A survival curve of HN1 in HCC. The data (mean ± standard deviation) between two groups were compared using unpaired *t* test. *P < .05, vs adjacent normal tissues. The experiment was repeated three times

The GSE94508 dataset obtained from National Center for Biotechnology Information (NCBI) showed hsa_circ_0000092 was highly expressed in human HCC tissues with |log2FC| > 1.5 and adj.*P*. Value (*P* value after correction) < .05 as the threshold. The Circular RNA Interactome (https://circinteractome.nia.nih.gov/index.html) further predicted that hsa_circ_0000092 might regulate the development of HCC. In addition, RT‐qPCR confirmed the differential expression of hsa‐miR‐188‐3p, hsa‐miR‐338‐3p, hsa‐miR‐513a‐5p and hsa‐miR‐587 between HCC and adjacent normal tissues and miR‐338‐3p was also predicted to interact with hsa_circ_0000092 regulating the progression of HCC (Figure 1A). Additionally, the starbase (http://starbase.sysu.edu.cn/index.php) and targetscan (http://www.targetscan.org/vert_71/) databases were used to predict the target gene of miR‐338‐3p, which was intersected with the up‐regulated genes in the GSE45267 dataset (Figure 1B). The results showed that only HN1 might be a target gene of miR‐338‐3p and its expression was elevated in the GSE45267 dataset (Figure 1C). Furthermore, the TCGA database (http://ualcan.path.uab.edu/index.html) was applied to analyse the potential regulatory genes in the development of HCC, which displayed that HN1 expression was increased in HCC and was correlated with the prognosis of HCC (Figure 1D,E). These results showed the possible involvement of hsa_circ_0000092, miR‐338‐3p and HN1 in the progression of HCC.

### 3.2 miR‐338‐3p is poorly expressed while hsa_circ_0000092 and HN1 are highly expressed in HCC tissues and cell lines

**FIGURE 2:**
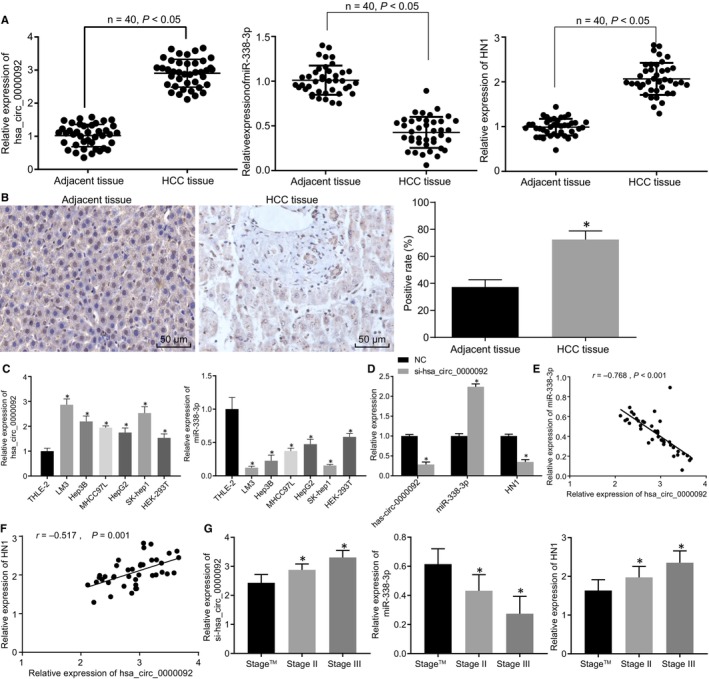
HCC tissues and cells exhibit up‐regulated hsa_circ_0000092 and HN1 but down‐regulated miR‐338‐3p. A, Expression of hsa_circ_0000092, miR‐338‐3p and HN1 in HCC and adjacent normal tissues measured by RT‐qPCR. B, The positive expression of HN1 in HCC and adjacent normal tissues determined by IHC (original magnification ×200). C, Expression of hsa_circ_0000092 and miR‐338‐3p in cell lines (Hep3B, LM3, MHCC97L, SK‐hep1, HepG2 and THLE‐2), as measured by RT‐qPCR. D, Expression of hsa_circ_0000092, miR‐338‐3p and HN1 in LM3 cells treated with NC or si‐hsa_circ_0000092 determined by RT‐qPCR. E, Correlation between hsa_circ_0000092 and miR‐338‐3p expression analysed using Pearson's correlation analysis. F, Correlation between hsa_circ_0000092 and HN1 expression analysed using Pearson's correlation analysis. G, Analysis of the relationship between the expression of hsa_circ_0000092, miR‐338‐3p, HN1 and the clinicopathological characteristics of patients with HCC. In panel A and B, *P < .05, vs adjacent normal tissues; the data (mean ± standard deviation) between two groups were analysed using paired *t* test. n = 40. In panel C, *P < .05, vs THLE‐2 cells; data among multiple groups were analysed using one‐way ANOVA, followed by Tukey's post hoc test; n = 40. In panel D, *P < .05, vs LM3 cells treated with NC; data between two groups were analysed using unpaired *t* test; In panel G, *P < .05, vs stage I. The experiment was repeated three times

RT‐qPCR results revealed that the expression of hsa_circ_0000092 and HN1 was higher while that of miR‐338‐3p was lower in HCC tissues than in adjacent normal tissues (Figure 2A). IHC detected higher positive expression of HN1 in HCC tissues compared with adjacent normal tissues (Figure 2B). Additionally, RT‐qPCR was carried out to measure the expression of hsa_circ_0000092 and miR‐338‐3p in Hep3B, LM3, MHCC97L, SK‐hep1, HepG2 and THLE‐2 cell lines. The results indicated that hsa_circ_0000092 expression was higher while miR‐338‐3p expression was lower in LM3 and SK‐hep1 cells than the remaining cells (*P* < .05) (Figure 2C). Therefore, LM3 cells were selected for subsequent experiments. We found miR‐338‐3p expression was increased, and HN1 expression was decreased in LM3 cells in response to si‐hsa_circ_0000092 treatment (Figure 2D). Pearson's correlation analysis showed that hsa_circ_0000092 negatively correlated with miR‐338‐3p and positively correlated with HN1 (Figure 2E,F). Further analysis of the relationship between the expression of hsa_circ_0000092, miR‐338‐3p and HN1 with the clinicopathological characteristics of patients with HCC showed that hsa_circ_0000092 expression was positively correlated with tumour node metastasis (TNM) stage and metastasis, but was irrelated with other characteristics (age, histological grade and gender) (Table 2). Moreover, the higher TNM stage reflected higher expression of hsa_circ_0000092 and HN1, yet lower miR‐338‐3p expression (Figure 2G). Overall, HCC tissues and cell lines displayed elevated expression of hsa_circ_0000092 and HN1 but reduced expression of miR‐338‐3p.

**TABLE 2:** Correlation between hsa_circ_0000092 expression and clinicopathological characteristics of patients with HCC

Clinicopathological characteristics	Expression		Total	χ^2^	*P* value
	Low	High			
Age (y)				0.476	.490
≤60	15	13	28		
>60	5	7	12		
Histological grade				1.600	.260
Low	8	12	20		
High	12	8	20		
Sex				0.476	.490
Female	7	5	12		
Male	13	15	28		
TNM stage				21.541	.001
Ι/II	20	6	26		
III/IV	0	14	14		
N stage				0.102	.749
N0	8	9	17		
N1/N2/N3	12	11	23		
M				0.100	.752
M0	11	10	21		
M1	9	10	19		

Abbreviations: NCC, hepatocellular carcinoma; TNM, tumour node metastasis.

### 3.3 hsa_circ_0000092 binds to miR‐338‐3p

**FIGURE 3:**
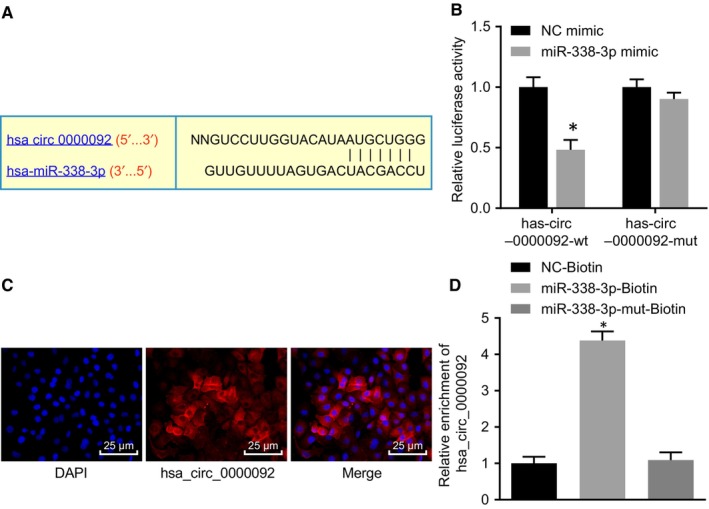
hsa_circ_0000092 competitively binds to miR‐338‐3p in LM3 cells. A, Binding sites between hsa_circ_0000092 and miR‐338‐3p analysed using the online prediction software. B, Interaction between hsa_circ_0000092 and miR‐338‐3p in LM3 cells verified by dual‐luciferase reporter assay. C, Localization of hsa_circ_0000092 in LM3 cells verified by FISH assay (Original magnification ×400). D, Enrichment of hsa_circ_0000092 detected using RNA pull‐down assay. *P < .05, vs treatment of NC mimic or NC‐biotin. The data (mean ± standard deviation) between two groups were analysed using unpaired *t* test, and data among multiple groups were analysed using one‐way ANOVA, followed by Tukey's post hoc test. The experiment was repeated three times

The online prediction software RNA22 (https://cm.jefferson.edu/rna22/) was used for bioinformatic analysis, and the results indicated that the hsa_circ_0000092 sequence contained a fragment that was complementary to miR‐338‐3p (Figure 3A). The interaction between hsa_circ_0000092 and miR‐338‐3p was further verified in LM3 and SK‐hep1 cells. First, we conducted the dual‐luciferase report assay and found that miR‐338‐3p mimic significantly reduced the luciferase activity of hsa_circ_0000092‐wt (Figure 3B; Figure S1A). In addition, the results of FISH showed that hsa_circ_0000092 was mainly localized in the cytoplasm (Figure 3C; Figure S1B). Furthermore, RNA pull‐down assay showed that miR‐338‐3p‐biotin could promote the enrichment of hsa_circ_0000092 (Figure 3D; Figure S1C). These results indicated that hsa_circ_0000092 could competitively bind to miR‐338‐3p.

### 3.4 hsa_circ_0000092 stimulates cell proliferation, migration, invasion and angiogenesis in HCC by binding to miR‐338‐3p

**FIGURE 4:**
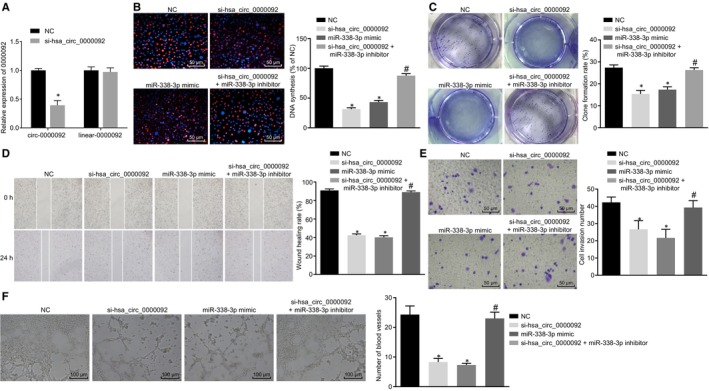
hsa_circ_0000092 promotes LM3 cell proliferation, invasion, migration and angiogenesis by binding to miR‐338‐3p. A, Expression of circ_0000092 and linear_0000092 in LM3 cells in response to NC treatment and si‐hsa_circ_0000092 treatment, as determined by RT‐qPCR. B, Representative views of EdU staining (original magnification ×200) and DNA synthesis of LM3 cells treated with NC, miR‐338‐3p mimic, si‐hsa_circ_0000092, or combined si‐hsa_circ_0000092 and miR‐338‐3p inhibitor, as determined using EdU assay. C, Colony formation rate of LM3 cells treated with NC, miR‐338‐3p mimic, si‐hsa_circ_0000092, or combined si‐hsa_circ_0000092 and miR‐338‐3p inhibitor, as determined by colony formation assay. D, Migration ability of LM3 cells treated with NC, miR‐338‐3p mimic, si‐hsa_circ_0000092, or combined si‐hsa_circ_0000092 and miR‐338‐3p inhibitor, as measured by scratch test. E, Invasion ability of LM3 cells treated with NC, miR‐338‐3p mimic, si‐hsa_circ_0000092, or combined si‐hsa_circ_0000092 and miR‐338‐3p inhibitor, as detected using Transwell assay (original magnification ×200). F, Representative views of blood vessels (original magnification ×100) and angiogenesis ability of LM3 cells treated with NC, miR‐338‐3p mimic, si‐hsa_circ_0000092, or combined si‐hsa_circ_0000092 and miR‐338‐3p inhibitor, as detected using vessel‐like tube formation in vitro. The data (mean ± standard deviation) among multiple groups were analysed using one‐way ANOVA, followed by Tukey's post hoc test. *P < .05, vs LM3 cells treated with NC; #*P* < .05, vs LM3 cells treated with si‐hsa_circ_0000092. The experiment was repeated three times

In order to demonstrate that it was the circular form rather than linear form of hsa_circ_0000092 that mediated the expression of miR‐338‐3p, the expression of circ_0000092 and linear_0000092 in LM3 cells was measured using RT‐qPCR. The results showed that si‐hsa_circ_0000092 treatment decreased the expression of circ_0000092 but showed no significant influence on the expression of linear_0000092 (Figure 4A), suggesting that siRNA exerted inhibitory effects on circ_0000092 expression in HCC. As shown by EdU assay, either si‐hsa_circ_0000092 or miR‐338‐3p mimic reduced LM3 cell viability; however, the cell viability was elevated in response to dual treatment of si‐hsa_circ_0000092 and miR‐338‐3p inhibitor relative to treatment of si‐hsa_circ_0000092 alone (Figure 4B).

The subsequent results of colony formation assay, scratch test and Transwell assay revealed that the LM3 cells transfected with either si‐hsa_circ_0000092 or miR‐338‐3p mimic exhibited decreased colony formation rate, migration and invasion abilities in contrast to the LM3 cells transfected with NC. Compared with si‐hsa_circ_0000092 treatment, co‐treatment of si‐hsa_circ_0000092 and miR‐338‐3p inhibitor increased colony formation rate, enhanced migration and invasion abilities (Figure 4C‐E).

Furthermore, the culture medium of LM3 cell lines transfected with NC, si‐hsa_circ_0000092, miR‐338‐3p mimic, miR‐338‐3p inhibitor, or combined si‐hsa_circ_0000092 and miR‐338‐3p inhibitor was delivered into the culture medium of HUVECs to co‐culture with vascular endothelial cells to conduct vessel‐like tube formation in vitro. The results indicated that either si‐hsa_circ_0000092 or miR‐338‐3p mimic treatment attenuated tube formation ability. The tube formation ability was enhanced by combined treatment of si‐hsa_circ_0000092 and miR‐338‐3p inhibitor relative to the treatment of si‐hsa_circ_0000092 alone (Figure 4F). Additionally, the above‐mentioned experiments were repeated in SK‐hep1 cells and the results obtained were consistent with that from LM3 cells (Figure S2). Therefore, hsa_circ_0000092 promoted proliferation, invasion, migration and angiogenesis abilities of HCC cells by down‐regulating miR‐338‐3p.

### 3.5 hsa_circ_0000092 up‐regulates the expression of HN1 by binding to miR‐338‐3p

**FIGURE 5:**
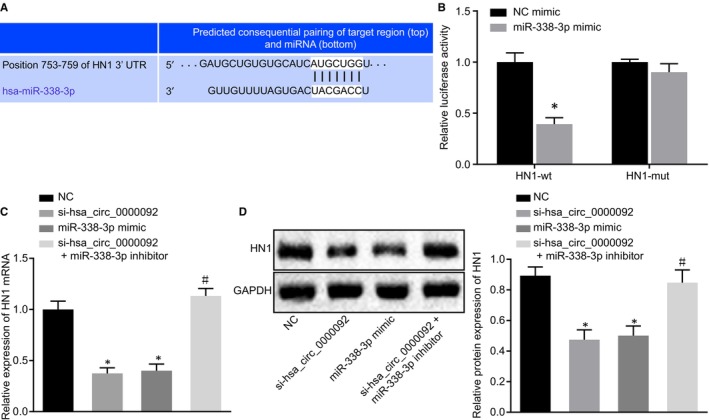
hsa_circ_0000092 up‐regulates HN1 expression by competitively binding to miR‐338‐3p in LM3 cells. A, Binding sites between miR‐338‐3p and HN1 predicted using the online bioinformatic prediction software. B, Luciferase activity of LM3 cells following co‐treatment of miR‐338‐3p and HN1‐3′UTR‐wt or co‐treatment of miR‐338‐3p and HN1‐3′UTR‐mut. C, mRNA expression of HN1 in LM3 cell lines transfected with NC, si‐hsa_circ_0000092, miR‐338‐3p mimic, or combined si‐hsa_circ_0000092 and miR‐338‐3p inhibitor, detected by RT‐qPCR. D, Protein expression of HN1 in LM3 cell lines transfected with NC, si‐hsa_circ_0000092, miR‐338‐3p mimic, or combined si‐hsa_circ_0000092 and miR‐338‐3p inhibitor, determined by Western blot analysis. The data (mean ± standard deviation) between two groups were tested using unpaired *t* test, and data among multiple groups were analysed using one‐way ANOVA and subjected to Tukey's post hoc test. *P < .05, vs LM3 cells treated with NC or NC mimic; #*P* < .05, vs LM3 cells treated with si‐hsa_circ_0000092. The experiment was repeated three times

The online prediction software, RNA22 (https://cm.jefferson.edu/rna22/), identified a binding site between miR‐338‐3p and HN1 (Figure 5A). The results of dual‐luciferase reporter assay confirmed that the luciferase activity of HN1‐3′UTR‐wt was reduced in LM3 and SK‐hep1 cells following transfection with miR‐338‐3p mimic while the luciferase activity of HN1‐3′UTR‐mut was not affected (Figure 5B; Figure S3A). Moreover, RT‐qPCR and Western blot analysis were conducted to measure the mRNA and protein expression of HN1 in LM3 and SK‐hep1 cell lines transfected with NC, si‐hsa_circ_0000092, miR‐338‐3p mimic, or combined si‐hsa_circ_0000092 and miR‐338‐3p inhibitor, respectively. Compared with NC treatment, treatment with miR‐338‐3p mimic or si‐hsa_circ_0000092 reduced the expression of HN1. In comparison to si‐hsa_circ_0000092 treatment, the expression of HN1 was increased following co‐treatment of si‐hsa_circ_0000092 and miR‐338‐3p inhibitor (Figure 5C,D; Figure S3B,C). Taken together, hsa_circ_0000092 competitively binds to miR‐338‐3p to elevate the expression of HN1 in HCC cells.

### 3.6 miR‐338‐3p negatively regulates HN1 to inhibit cell proliferation, migration, invasion and angiogenesis in HCC

**FIGURE 6:**
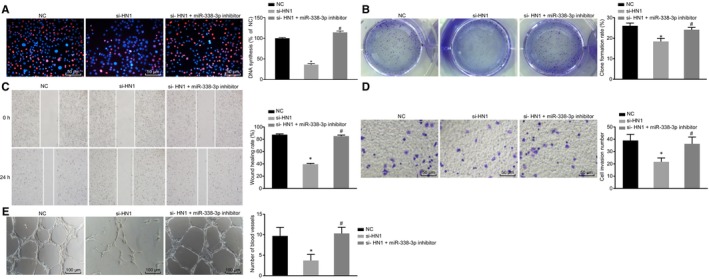
miR‐338‐3p overexpression represses the proliferation, migration, invasion and angiogenesis of HCC cells by targeting HN1. A, Representative views of EdU staining (original magnification ×200) and DNA synthesis of LM3 cells treated with NC, si‐HN1, or combined si‐HN1 and miR‐338‐3p inhibitor. B, Colony formation ability of LM3 cells treated with NC, si‐HN1, or combined si‐HN1 and miR‐338‐3p inhibitor, as measured by colony formation assay. C, Migration ability of LM3 cells treated with NC, si‐HN1, or combined si‐HN1 and miR‐338‐3p inhibitor, as detected by scratch test. D, Invasion ability of LM3 cells treated with NC, si‐HN1, or combined si‐HN1 and miR‐338‐3p inhibitor, as detected by Transwell assay (original magnification ×200). E, Representative views of blood vessels (original magnification ×100) and angiogenesis ability of LM3 cells treated with NC, si‐HN1, or combined si‐HN1 and miR‐338‐3p inhibitor, as assessed by vessel‐like tube formation in vitro. The data (mean ± standard deviation) among multiple groups were analysed using one‐way ANOVA and subjected to Tukey's post hoc test. *P < .05, vs LM3 cells treated with NC; #*P* < .05, vs LM3 cells treated with si‐HN1. The experiment was repeated three times

To further explore the functional role of miR‐338‐3p in biological characteristics with the involvement of HN1, EdU assay, colony formation assay, scratch test and Transwell assay were performed. The results obtained showed that cell viability, colony formation rate, migration and invasion abilities of LM3 cells were reduced in response to treatment of si‐HN1 compared with NC treatment. In contrast to si‐HN1 treatment, combined treatment with si‐HN1 and miR‐338‐3p inhibitor increased cell viability, colony formation rate, and migration and invasion abilities of LM3 cells (Figure 6A‐D). Furthermore, culture medium of LM3 cells treated with NC, si‐HN1, or combined si‐HN1 and miR‐338‐3p inhibitor was added to the culture medium of HUVECs to conduct vessel‐like tube formation in vitro. The results suggested that the tube formation ability of si‐HN1‐treated LM3 cells was reduced compared with NC treatment, while tube formation ability was enhanced by the co‐treatment of si‐HN1 and miR‐338‐3p inhibitor when compared with si‐HN1 treatment (Figure 6E). These findings provided evidence that miR‐338‐3p‐mediated HN1 inhibition decreases the proliferation, migration, invasion and angiogenesis of HCC cells.

### 3.7 hsa_circ_0000092 silencing hinders the progression of HCC in vivo

To assess the effects of hsa_circ_0000092 on tumour growth in vivo, LM3 cells treated with si‐hsa_circ_0000092 were utilized to prepare a cell suspension, which was then subcutaneously injected into nude mice. The results illustrated that the mice injected with cells expressing si‐hsa_circ_0000092 had prolonged survival (Figure S4A). In addition, the expression of hsa_circ_0000092 was measured using ISH assay, which showed that expression of hsa_circ_0000092 was down‐regulated after treatment of si‐hsa_circ_0000092 (Figure S4B). Moreover, the average tumour volume and weight were reduced in mice treated with si‐hsa_circ_0000092 (Figure S4C‐E). Furthermore, IHC showed reduced positive expression of HN1, PCNA, MMP2, MMP9 and VEGF proteins in response to the delivery of si‐hsa_circ_0000092 expressing cells (Figure S4F). Based on these findings, we reasoned that depletion of hsa_circ_0000092 could suppress the development of HCC in vivo.

## 4 DISCUSSION

Increasing evidence has shown that the circRNA‐miR‐mRNA axis is highly involved in the progression of HCC and can provide new insights into the specific mechanisms of HCC progression as well as help to identify biomarkers and targets for HCC treatment.^20^, ^21^ In this study, we utilized microarray analysis to screen the differentially expressed HCC‐related circRNAs and predicted their underlying function in HCC development, finding that the hsa_circ_0000092/miR‐338‐3p/HN1 axis was associated with HCC progression. Thus, in vitro and in vivo assays were performed to identify the potential effects of this axis on HCC. The results demonstrated that silencing hsa_circ_0000092 could down‐regulate HN1 and inhibit HCC development via up‐regulation of miR‐338‐3p.

Our initial results showed that the expression of hsa_circ_0000092 and HN1 was higher while that of miR‐338‐3p was lower in HCC tissues and cell lines. CircRNAs are covalently closed loop single RNAs, predominantly found in the cytoplasm and showing important functions in tissue development and epithelial–mesenchymal transition (EMT).^22^ Consistent with our findings, hsa_circ_0128298 is also highly expressed in HCC tissues and is associated with the enhanced proliferation and metastasis of HCC cells, thus acting as a potential diagnostic and prognostic marker for patients with HCC.^23^ Similarly, Guan et al^24^ showed that HCC tissues and cells exhibited overexpressed hsa_circ_0016788 and that down‐regulation of hsa_circ_0016788 repressed HCC development; hence, hsa_circ_0016788 was proposed as an oncogene in HCC. HN1 is widely recognized as a gene encoding a protein that is extensively expressed in a variety of tissues.^25^, ^26^ In line with our study, HN1 has been found to be markedly up‐regulated in HCC tissues relative to the adjacent normal tissues and its high expression has been significantly associated with a poor overall survival of patients with HCC.^16^ Also, HN1 could be used for prognosticating HCC due to its association with disease‐specific survival of patients with HCC.^27^ However, the regulation of HN1 by circRNAs has not been reported. In the study, we found the bridge regulator between hsa_circ_0000092 and HN1 that was miR‐338‐3p. In agreement with our findings, miR‐338‐3p has been revealed to be down‐regulated in metastatic HCC tissues and cells and the expression of miR‐338‐3p is related to low metastasis‐free survival, suggesting miR‐338‐3p could potentially serve as an underlying biomarker for the treatment of HCC patients with metastasis.^28^ Likewise, forced expression of miR‐338‐3p can inhibit HCC cell proliferation and colony formation while inducing cell cycle arrest, indicating that miR‐338‐3p exerts a tumour suppressive role in HCC development.^29^ Therefore, we reasoned the regulatory axis of hsa_circ_0000092/miR‐338‐3p/HN1 was associated with HCC.

CircRNAs could act as sponges or ceRNAs of miRNAs, regulating the expression of miRs and participating in the development of diseases.^30^, ^31^ Partly in line with our findings, hsa_circ_0000673, which is overexpressed in HCC tissues, could serve as a sponge of miR‐767‐3p to down‐regulate miR‐767‐3p expression and depletion of hsa_circ_0000673 could hinder the progression of HCC.^32^ Moreover, a growing number of studies suggest that lncRNAs regulate expression of mRNAs by competitively binding to miRNAs and thus contributing to the development of diseases.^33^ Circ‐PDE8A up‐regulates the expression of MACC1 and then accelerates pancreatic ductal adenocarcinoma progression by functioning as a ceRNA of miR‐338.^34^ In addition, silencing of hsa_circ_000984 down‐regulates the expression of CDK4 by competitively binding with miR‐106b, thus hindering cellular processes in vitro and tumour growth in vivo in colorectal cancer.^35^ Emerging evidence demonstrates that miRNAs play a crucial role in the initiation and progression of cancer through their regulatory roles in cell growth, invasion and metastasis by inhibiting the expression of their targets.^36^, ^37^ miR‐338‐3p has been revealed to bind to the smoothened gene to further inhibit cell invasion in liver cancer cells, suggesting that miR‐338‐3p is an underlying biomarker for the management of liver cancer.^12^ Our current study revealed that miR‐338‐3p could curb HCC cell proliferation, migration, invasion and angiogenesis by inhibiting its target HN1. On the basis of the aforementioned, our studies reveal a previously unrecognized hsa_circ_0000092/miR‐338‐3p/HN1 signalling axis in HCC.

Taken together, depletion of hsa_circ_0000092 decreases the expression of HN1 and consequently hinders the progression and development of HCC via up‐regulation of miR‐338‐3p (Figure S5). Our study intriguingly highlights the potential of hsa_circ_0000092, HN1 and miR‐338‐3p as novel therapeutic targets for the treatment of patients with HCC and may contribute to deeper exploration of the specific mechanisms of circRNA‐miR‐mRNA network in cancers. In our future study, we will combine the silencing hsa_circ_0000092 and HN1, and overexpressing miR‐338‐3p with traditional chemotherapy drugs to elucidate the synergetic effects on HCC retardation and to provide a basis for further research of related inhibitors.

## CONFLICT OF INTEREST

None.

## AUTHOR CONTRIBUTION

Jianchu Wang, Wenchuan Li, Yuan Lu and Xianjian Wu designed the study. Xidai Long, Chunying Luo and Huamei Wei collated the data, carried out data analyses and produced the initial draft of the manuscript. Jian Pu contributed to drafting the manuscript. All authors have read and approved the final submitted manuscript.

## Supporting information

 

 

 

 

 

 

## Data Availability

The datasets generated and/or analysed during the current study are available from the corresponding author on reasonable request.
