# Supplementary material for: hsa_circ_0000092 promotes hepatocellular carcinoma progression through up‐regulating HN1 expression by binding to microRNA‐338‐3p
Source: J Cell Mol Med. 2020 Feb 20;28(6):e15010. doi: 10.1111/jcmm.15010 (PMC10941524; doi:10.1111/jcmm.15010)
Supplement: Supplementary file 6 [file JCMM-28-e15010-s004.docx]

**Supplementary Figure S1.** hsa_circ_0000092 competitively binds to miR-338-3p in SK-hep1 cells. A, Interaction between hsa_circ_0000092 and miR-338-3p in SK-hep1 cells verified by dual-luciferase reporter assay. B, Localization of hsa_circ_0000092 in SK-hep1 cells verified by FISH assay (× 400). C, Enrichment of hsa_circ_0000092 detected using RNA pull-down assay. * *p* < 0.05, *vs.* treatment of NC mimic or NC-biotin. The data (mean ± standard deviation) between two groups were analysed using unpaired *t* test, and data among multiple groups were analysed using one-way ANOVA, followed by Tukey’s post hoc test. The experiment was repeated three times.

**Supplementary Figure S2.** hsa_circ_0000092 stimulates SK-hep1 cell proliferation, invasion, migration and angiogenesis by binding to miR-338-3p. A, Relative expression of circ_0000092 and linear_0000092 in SK-hep1 cells in response to NC treatment and si-hsa_circ_0000092 treatment, as determined by RT-qPCR. B, Representative views of EdU staining (Original magnification × 200) and DNA synthesis of SK-hep1 cells treated with NC, miR-338-3p mimic, si-hsa_circ_0000092, or combined si-hsa_circ_0000092 and miR-338-3p inhibitor, as determined using EdU assay. C, Colony formation rate of SK-hep1 cells treated with NC, miR-338-3p mimic, si-hsa_circ_0000092, or combined si-hsa_circ_0000092 and miR-338-3p inhibitor, as determined by colony formation assay. D, Migration ability of SK-hep1 cells treated with NC, miR-338-3p mimic, si-hsa_circ_0000092, or combined si-hsa_circ_0000092 and miR-338-3p inhibitor, as measured by scratch test. E, Invasion ability of SK-hep1 cells treated with NC, miR-338-3p mimic, si-hsa_circ_0000092, or combined si-hsa_circ_0000092 and miR-338-3p inhibitor, as detected using Transwell assay (Original magnification × 200). F, Representative views of blood vessels (original magnification × 100) and angiogenesis ability of SK-hep1 cells treated with NC, miR-338-3p mimic, si-hsa_circ_0000092, or combined si-hsa_circ_0000092 and miR-338-3p inhibitor, as detected using vessel-like tube formation *in vitro*. The data (mean ± standard deviation) among multiple groups were analysed using one-way ANOVA, followed by Tukey’s post hoc test. * *p* < 0.05, *vs.* SK-hep1 cells treated with NC; # *p* < 0.05, *vs.* SK-hep1 cells treated with si-hsa_circ_0000092. The experiment was repeated three times.

**Supplementary Figure S3.** hsa_circ_0000092 up-regulates HN1 expression by competitively binding to miR-338-3p in SK-hep1 cells. A, Binding sites between miR-338-3p and HN1 predicted using online bioinformatic prediction software. B, Luciferase activity of SK-hep1 cells following the co-treatment of miR-338-3p and HN1-3’UTR-wt or co-treatment of miR-338-3p and HN1-3’UTR-mut. C, mRNA expression of HN1 in SK-hep1 cell lines manipulated with NC, si-hsa_circ_0000092, miR-338-3p mimic, or combined si-hsa_circ_0000092 and miR-338-3p inhibitor, detected by RT-qPCR. D, Protein expression of HN1 in SK-hep1 cell lines manipulated with NC, si-hsa_circ_0000092, miR-338-3p mimic, or combined si-hsa_circ_0000092 and miR-338-3p inhibitor, determined by Western blot analysis. The data (mean ± standard deviation) between two groups were tested using unpaired *t* test, and data among multiple groups were analysed using one-way ANOVA and subjected to Tukey’s post hoc test. * *p* < 0.05, *vs.* SK-hep1 cells treated with NC or NC mimic; # *p* < 0.05, *vs.* SK-hep1 cells treated with si-hsa_circ_0000092. The experiment was repeated three times.

**Supplementary Figure S4.** The development of HCC *in vivo* could be mitigated by the down-regulation of hsa_circ_0000092. A, Survival rate of the mice injected with NC or si-hsa_circ_0000092. B, Expression of hsa_circ_0000092 in response to treatment of NC or si-hsa_circ_0000092 detected by ISH (original magnification × 200). C, Representative images of xenograft tumours in mice injected with NC or si-hsa_circ_0000092. D, Tumour volume of mice injected with NC or si-hsa_circ_0000092. E, Tumour weight of mice injected with NC or si-hsa_circ_0000092. F, Representative images of HN1-, PCNA-, MMP2-, MMP9- and VEGF-positive expression in mice injected with NC or si-hsa_circ_0000092, as measured by IHC (Original magnification × 200). * *p* < 0.05, *vs.* the mice injected with NC. The data (mean ± standard deviation) between two groups in panel A and F were tested using unpaired *t* test, data in panel D were analysed using repeated measures ANOVA and subjected to Tukey’s post hoc test, and data in panel E were analysed using unpaired *t* test. n = 15.

**Supplementary Figure S5.** Potential regulatory mechanism of hsa_circ_0000092 in the development of HCC by regulating miR-338-3p-mediated HN1. hsa_circ_0000092 and HN1 were highly expressed while miR-338-3p was poorly expressed in HCC. Blocked hsa_circ_0000092 could up-regulate the expression of miR-338-3p to deplete the expression of HN1, thus suppressing the proliferation, migration, invasion and angiogenesis of HCC cells *in vitro* as well as the tumour growth of HCC *in vivo*.
